# Integrated metabolomic and transcriptomic analysis elucidates transcriptional regulation of flavonoid biosynthesis in differentially pigmented honeysuckle (*Lonicera japonica*) varieties

**DOI:** 10.3389/fpls.2025.1636028

**Published:** 2025-09-11

**Authors:** Hailei Liao, Fang Wu, Jixin Xie, Wenying He, Xinxue Zhang, Jinxia Dai, Hua Liu, Ming Li, Lijuan Wang

**Affiliations:** ^1^ College of Basic Medical Science, Ningxia Medical University, Yinchuan, China; ^2^ Guyuan Branch of Ningxia Academy of Agricultural and Forestry Sciences, Guyuan, China; ^3^ Life College, Ningxia University, Yinchuan, China; ^4^ Institute of Forestry and Grassland Ecology, NingXia Academy of Agriculture and Forestry Sciences, Yinchuan, China

**Keywords:** *Lonicera japonica*, flavonoid biosynthesis, transcriptomics, metabolomics, anthocyanins, MYB transcription factors

## Abstract

**Introduction:**

Honeysuckle (*Lonicera japonica* Thunb.) is a key medicinal plant whose bioactive flavonoids underpin its antiviral, anti-inflammatory, and antioxidant properties. However, the genetic and metabolic mechanisms controlling flavonoid accumulation and the associated flower color variation between different cultivars remain largely unexplored, limiting targeted breeding for enhanced therapeutic quality.

**Methods:**

A comparative analysis was conducted using two distinct honeysuckle varieties—'Luyu No.1' (white-yellow flowers) and 'Honghua' (red-pink flowers)—at the optimal harvest stage ('Dabai period'). An integrated transcriptomics (RNA-seq) and widely targeted metabolomics approach was employed to identify differentially expressed genes (DEGs) and differentially accumulated metabolites (DAMs) between the varieties.

**Results:**

Transcriptomic profiling revealed 5,901 DEGs, with significant enrichment in the phenylpropanoid and flavonoid biosynthesis pathways. Key structural genes (PAL, CHS, F3H, FLS, DFR, and ANS) were markedly up-regulated in 'Honghua'. Metabolomic analysis identified 399 flavonoids, including 228 DAMs. The 'Honghua' variety exhibited a 2.32-fold higher total flavonoid content, with substantially elevated levels of anthocyanidins (87.58-fold), flavanols (7.66-fold), and chalcones (6.17-fold). Critical medicinal compounds such as quercetin-3-O-rhamnoside and luteolin-7-O-glucoside were significantly enriched in 'Honghua'. Integrated analysis highlighted the co-expression of transcription factors (MYB, bHLH, NAC) with flavonoid pathways. Crucially, the anthocyanin-modifying enzymes F3’M and 3RT were up-regulated by 22.12- and 3.03-fold, respectively, in 'Honghua', while a potential repressor, TCP15 (bHLH), was down-regulated.

**Discussion:**

The results demonstrate that the red-pink pigmentation and superior medicinal quality of 'Honghua' are driven by a coordinated gene-metabolite network that fluxes metabolic intermediates towards anthocyanin and other flavonoid end-products. The pronounced up-regulation of F3’M and 3RT is identified as a key biochemical determinant for anthocyanin diversification and color development. This study provides the first comprehensive framework for flavonoid biosynthesis in honeysuckle, establishing a genetic basis for breeding cultivars with high flavonoid content and enhanced pharmacological value.

## 1 Introduction

Honeysuckle (*Lonicera japonica* Thunb.), a traditional medicinal plant from the Caprifoliaceae family, is primarily made from dried flower buds or early-blooming flowers (Law et al., 2024). Traditionally, it has been used for heat-clearing, detoxifying, and dispelling wind-heat (Huo et al., 2024), treating conditions like furuncles, carbuncles, sore throat, erysipelas, dysentery, and colds. Modern research shows honeysuckle has antiviral (Ma et al., 2024), anti-inflammatory, and antioxidant effects (Ma et al., 2025), earning its reputation as “antibiotics in traditional Chinese medicine.” It contains various compounds, such as volatile oils, flavonoids, organic acids, iridoid glycosides, and triterpene saponins, making it useful in clinical, pharmaceutical, health care, and cosmetic applications.

Honeysuckle, although not indigenous to the Ningxia Hui Autonomous Region, has been successfully cultivated there since 2021 on selenium-enriched sandy soil. Two major varieties, ‘Luyu No.1’ (characterized by white-yellow floral buds) and ‘Honghua’ (distinguished by red-pink floral buds), represent predominant types currently grown in the region and display marked differences in pigmentation. Notably, this variation in coloration is closely correlated with well-documented differences in their flavonoid composition, particularly anthocyanin levels—key bioactive compounds that contribute to honeysuckle’s pharmacological properties, including antioxidant and antiviral activities (Zheng et al., 2022). Therefore, these two varieties provide a valuable comparative system for investigating the molecular mechanisms governing flavonoid biosynthesis, accumulation patterns, and flower color differentiation, all of which have direct implications for medicinal efficacy. The ‘Dabai period’, corresponding to the stage of maximal accumulation of bioactive constituents, represents the optimal time for harvest. Honeysuckle buds are abundant in bioactive flavonoids (Li et al., 2024), such as rutin, luteolin, loniceroside, quercetin, and hyperoside, which exhibit a broad spectrum of therapeutic effects, including antioxidant, anti-inflammatory, antiviral, immunomodulatory, cardioprotective, and diuretic properties (Yang et al., 2023). It is well established that variations among honeysuckle varieties result in differences in the content of their bioactive components (Hou et al., 2025). However, the molecular basis underlying the differential accumulation of these flavonoids across cultivars remains poorly understood.

Advances in high-throughput sequencing and systems biology have made multiomics approaches essential for understanding plant growth and development at systemic and cellular levels (Li et al., 2023. Metabolomics analyzes small molecule metabolites qualitatively and quantitatively, while transcriptomics tracks gene expression changes. Integrating metabolomics and transcriptomics via ‘metabolite and gene’ co-expression analysis improves the elucidation of metabolic biosynthesis and regulation mechanisms and aids in identifying candidate genes (Liu et al., 2023). For example, Zhang et al. (2021) found increased activity of anthocyanin biosynthesis-related genes in jujube fruits, with kaempferol 3-O-glucoside and delphinidin 3-O-glucoside accumulation contributing to red peel coloration. Wu et al. (2016) combined transcriptomic and metabolomic analyses to identify key flavonoid pathway genes, revealing blue water lily flower color formation. Guo et al. (2019) linked differential gene expression in the flavonoid pathway to tea leaf color differences. Zhuang et al. (2019) showed that dihydroflavonols are transferred to anthocyanin biosynthesis, causing pigment differences in purple and green radish root peels.

In this study, ‘Luyu No.1’ and ‘Honghua’ honeysuckle varieties were analyzed using metabolomics to investigate flavonoid composition differences and their synthesis mechanisms. Transcriptomics was used to identify key genes and transcription factors related to flavonoid synthesis, exploring the genetic basis of flower color changes. The study aims to clarify the molecular mechanisms of flower color differences under consistent ecological conditions and identify critical genes involved in active component synthesis. These findings will provide a theoretical basis for anthocyanin synthesis research and breeding improvements, as well as support the development of gene-specific markers and targeted breeding strategies.

## 2 Materials and methods

### 2.1 Plant materials and growth conditions

**Figure 1:**
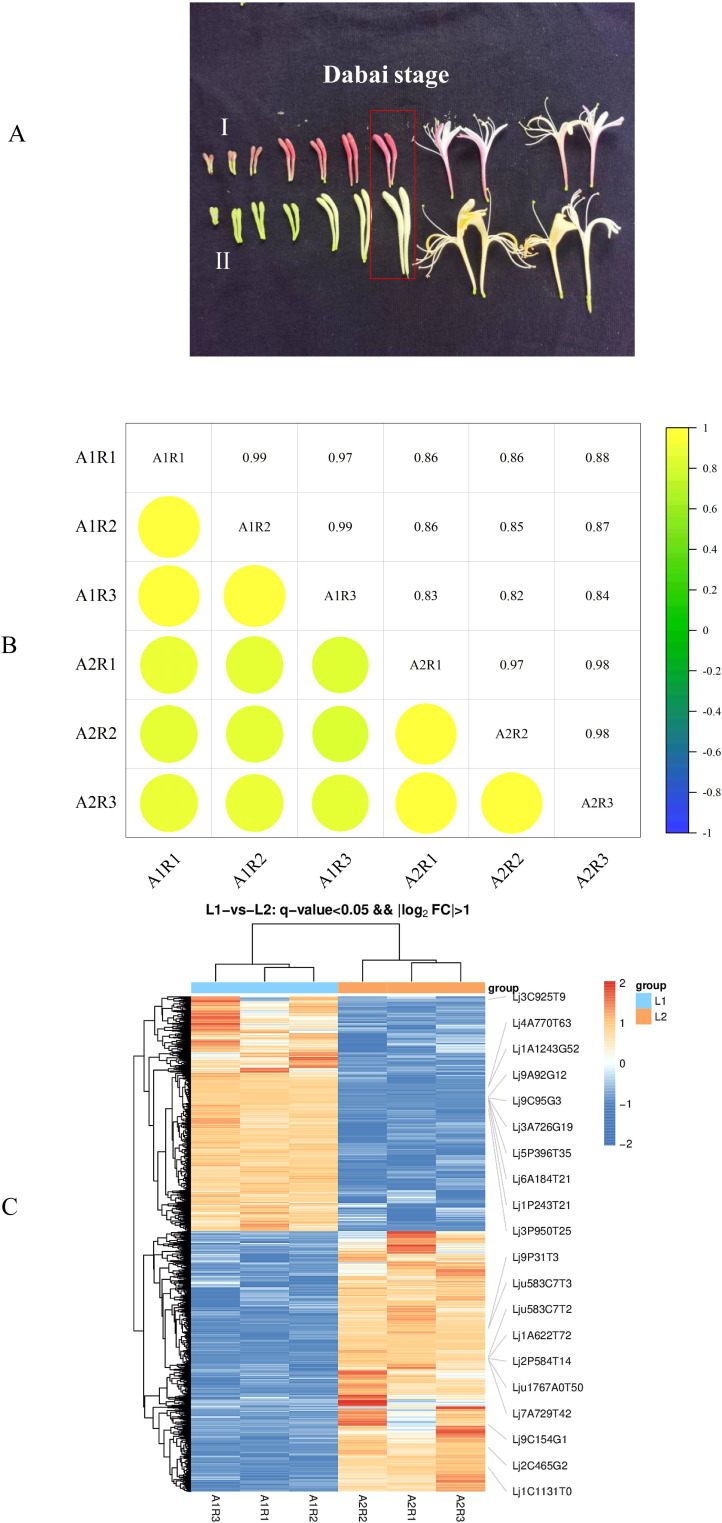
**(A)** Fresh flower appearance of ‘Honghua’(I) and ‘Luyu No.1’ (II), **(B)** An Individual correlation analyses between 6 transcriptome samples of ‘Honghua’ and ‘Luyu No.1’, **(C)** Volcano map of DEGs.

The *Lonicera japonica* Thunb. cultivars ‘Luyu No. 1’ and ‘Honghua’ were grown in the standardized field of the Ningxia honeysuckle planting base (37°20′11.053”N, 105°15′11.053”E). Flowers at the Dabai stage, characterized by stick-shaped buds before blooming (**Figure 1A**), were harvested using diagonal sampling. Three replicates, each from at least three plants, were collected. Samples were split into two groups: one for biochemical validation and the other frozen at -80 °C for sequencing and analysis.

### 2.2 RNA extraction and Illumina sequencing

Total RNA and mRNA extraction for sequencing was performed using Metwell Biotechnology Co., Ltd. (Wuhan, China). To ensure the RNA met the quality requirements for library construction, its purity, concentration, and integrity were assessed using the NanoPhotometer^®^ spectrophotometer (IMPLEN, CA, USA), the Qubit^®^ RNA Assay Kit in the Qubit^®^ 2.0 Fluorometer (Life Technologies, CA, USA), and the RNA Nano 6000 Assay Kit on the Bioanalyzer 2100 System (Agilent Technologies, CA, USA). The cDNA library was constructed using the cDNA Library Construction Kit (Beijing Genomics Institute, Shenzhen, China) and sequenced on the Illumina NovaSeq 6000 platform at Metwell Biotechnology Co., Ltd. (Wuhan, China).

### 2.3 Sequence annotation and classification

New transcript information was extracted from the comparison results of spliced transcripts and genome annotation, and then the sequence of new genes was collected from the genome. Using BlastX, the sequences were explored for annotation against the NCBI nonredundant (NR, http://www.ncbi.nlm.nih.gov) protein database with a cut-of E-value of 10^–5^. Blast2GO (version: 2.5.0, parameters: default) was used to retrieve Gene Ontology (GO) terms from the annotation of high scoring BLAST matches against the NCBI NR protein database (E-value ≤ 1.0×10^–5^, http://www.ncbi.nlm.nih.gov) Then, the GO categories were sorted with in-house Perl scripts. The Kyoto Encyclopedia of Genes and Genomes pathways (KEGG, http://www.genome.jp/kegg) were annotated against the KEGG database using Blast all software (version:2.2.23, parameters:default). In addition, the sequences were annotated by aligning them in the Cluster of Orthologous Groups of proteins (KOG, https://www.ncbi.nlm.nih.gov/COG/), and the manual annotation and reviewing of protein sequences were conducted with the SwissProt (http://www.expasy.ch/sprot) and Protein family databases (Pfam, https://www.ebi.ac.uk/interpro/entry/pfam/). The Plant Transcription Factor Database (Plant TFDB) and PlnTFDB were used to annotate and classify the transcription factors (TFs) using iTAK (v1.7a).

### 2.4 Analysis of differentially expressed genes

Using ‘Luyu No. 1’ flowers as the control, transcriptome data from ‘Honghua’ flowers were analyzed via high-throughput sequencing technology. The number of reads per gene was quantified based on alignment results and gene location information on the *Lonicera japonica* reference genome. Gene expression levels were calculated as fragments per kilobase of transcript per million fragments mapped (FPKM), normalized by gene length and mapped read count. Differential expression analysis between the two groups was performed using DESeq2 version 1.22.1. The false discovery rate (FDR) was estimated by adjusting p-values with the Benjamini- Hochberg method. Differentially expressed genes (DEGs) were identified using thresholds of |log2Fold Change| ≥ 1.5 and FDR < 0.05. Subsequently, GO function enrichment, KOG annotation, and KEGG pathway enrichment analyses were conducted for the DEGs.

### 2.5 Real−time quantitative RT-PCR analysis

Real-time quantitative PCR (RT-qPCR) was employed to validate the reliability of the RNA-seq results. Seven key candidate genes, which play critical roles in flavonoid metabolism, were selected for analysis. The same RNA samples used in the transcriptome analysis were reverse transcribed into cDNA using the HiScript^®^ II Q RT SuperMix for qPCR (+gDNA wiper) kit (Vazyme Biotech Co., Ltd., Nanjing, China). Primers were designed using Primer Premier 5.0 software and synthesized by Shanghai Shenggong Biotechnology Co., Ltd. The primer sequences are listed in **Supplementary Table S1**. The constitutively expressed honeysuckle Actin gene (GenBank: KY114518) was used as an internal control for normalizing gene expression levels. Real-time PCR was performed in a 20 μL reaction mixture containing 50 ng of template cDNA, 10 μL of 2× Universal SYBR Green Fast qPCR Mix (ABclonal Technology Co., Ltd., Wuhan, China), 0.4 μL of each primer, and ddH_2_O. Amplification was carried out on the qTOWER Real-Time PCR Instrument (Analytik Jena AG, Germany) under the following thermal cycling conditions: 95 °C for 3 min, followed by 40 cycles of 95 °C for 5 s and 60 °C for 30 s. Relative gene expression levels were calculated using the 2^−ΔΔCt^ method. To ensure reproducibility and reliability, three independent biological replicates per sample and three technical replicates per biological replicate were conducted.

### 2.6 Sample preparation and LC-MS

The flowers were freeze-dried (Scientz-100F) and ground into powder (Retsch MM 400). Approximately 50 mg of the powder was mixed with 1.2 mL of 70% methanol, vortexed for 30 s every 30 min over six cycles, and centrifuged at 12,000 rpm for 3 min. The supernatant was filtered (SCAA-104, 0.22 μm) and analyzed by UPLC-ESI-MS/MS (UPLC: Shimadzu Nexera X2; MS: Applied Biosystems 6500 Q TRAP). Conditions included an Agilent SB-C18 column (1.8 µm, 2.1 mm × 100 mm), mobile phase of water (A, 0.1% formic acid) and acetonitrile (B, 0.1% formic acid), and a gradient program starting at 95% A/5% B. Within 9 min, the gradient reached 5% A/95% B and was maintained for 1 min before reverting to 95% A/5% B within 1.1 min and held for 2.9 min. Flow rate was 0.35 mL/min, column temperature 40 °C, and injection volume 2 μL. ESI source parameters: temperature 500 °C, ion spray voltage 5500 V (positive)/-4500 V (negative), gases set to 50/60/25 psi, and CAD high.

### 2.7 Metabolome analysis

Principal component analysis (PCA) was performed using the PrComp function (v3.5.1). Differential metabolites were identified based on variable importance in projection (VIP ≥ 1) and absolute log2 fold change (|log2FC| ≥ 1.0). VIP values were derived from orthogonal partial least squares discriminant analysis (OPLS-DA) results using the R package MetaboAnalystR (v1.0.1). Prior to OPLS-DA, the data were preprocessed with log2 transformation and mean centering. To ensure model reliability and prevent overfitting, a permutation test was conducted with 200 permutations.

### 2.8 Comprehensive integrated analysis of transcriptional and metabolic profiles

Based on the identification of differentially expressed genes and metabolites involved in flavonoid metabolism, transcriptomic and metabolomic data were integrated and mapped onto the KEGG pathway diagram to further elucidate the relationships between these genes and their corresponding metabolites.

### 2.9 Statistical analysis

All experimental data were presented in triplicate. Data analysis was performed using SPSS 17.0 software. All data are expressed as mean ± standard deviation (SD). Comparisons between and within groups were conducted using Duncan’s multiple range test. Student’s t-test was applied to calculate P-values, with P < 0.05 indicating statistically significant differences.

## 3 Results

### 3.1 RNA-seq analysis

To summarize the transcriptomic differences between ‘Luyu No. 1’ and ‘Honghua’ flowers, six cDNA samples per type (three biological replicates each) were sequenced using Illumina NovaSeq6000. After quality control, 290.78 million clean reads were obtained, with Q30 values of 95.27%–96.88% and GC content of 44.97%–45.19%. The reads were aligned to the *Lonicera japonica* reference genome, resulting in mapping rates of 94.74%–95.91% (**Supplementary Table S2**). To assess the reliability of transcriptome sequencing data, Pearson correlation analysis was performed on flower samples of ‘Luyu No. 1’ and ‘Honghua’ during the Dabai stage. The correlation coefficient (0.82 < R² < 1, **Figure 1B**) indicated high gene homogeneity across the six samples. These results confirmed the high quality and integrity of the RNA-seq data, ensuring their suitability for subsequent analyses.

### 3.2 DEG analysis of ‘Luyu No. 1’ and ‘Honghua’

To identify DEGs, a comparative transcriptome analysis was conducted between ‘Luyu No. 1’ and ‘Honghua’ buds, with ‘Luyu No. 1’ as the control. A total of 5901 DEGs were detected (**Supplementary Table 2**), including 2799 up-regulated and 3102 down-regulated genes (**Figure 1C**), indicating significant differences in gene expression patterns.

**Figure 2:**
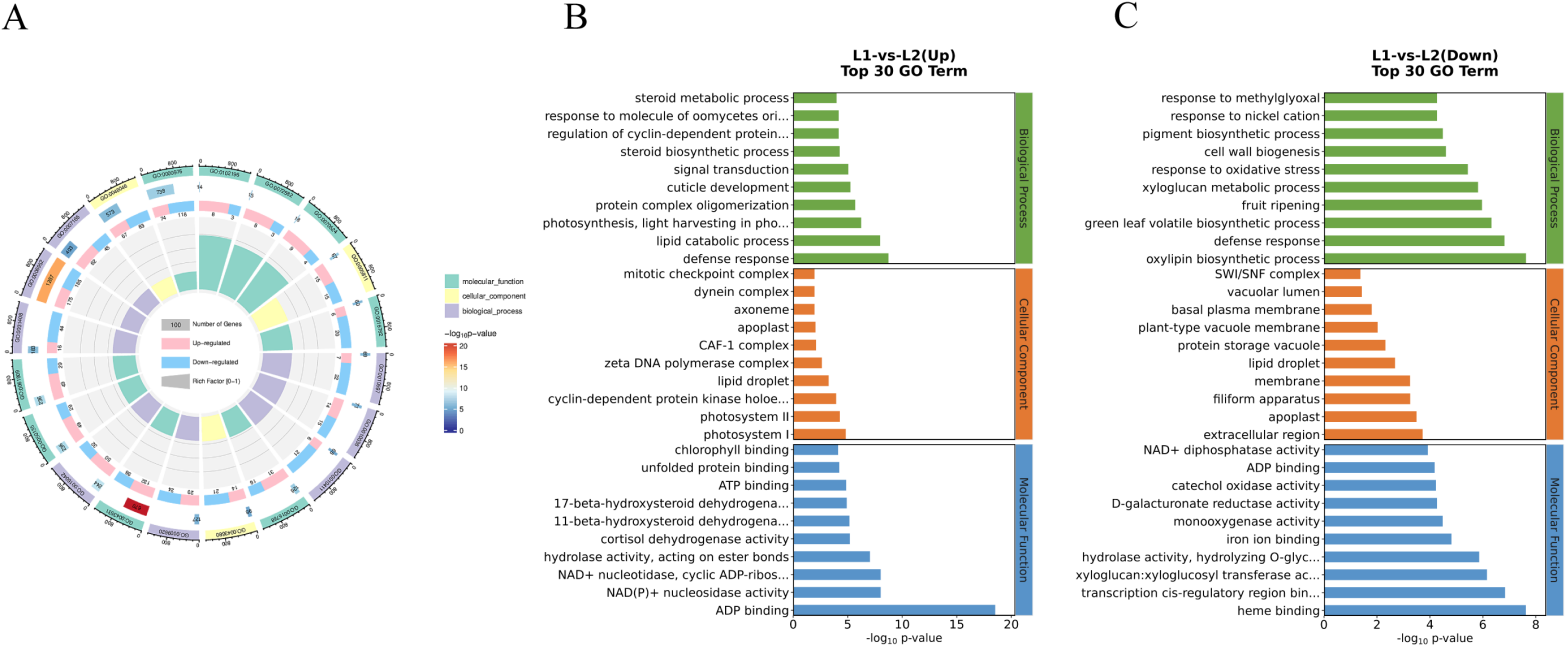
GO enrichment of DEGs. **(A)** Total DEGs of ‘Honghua’ and ‘Luyu No.1’, **(B)** Up DEGs of ‘Honghua’ and ‘Luyu No.1’, **(C)** Down DEGs of ‘Honghua’ and ‘Luyu No.1’.

The Gene Ontology (GO) database categorizes genes into three main groups: Cellular Component (CC), Biological Process (BP), and Molecular Function (MF). GO annotation revealed that DEGs were classified into these three categories. GO enrichment analysis further showed that among the top 20 enriched terms, defense response (1287 DEGs), ADP binding (738 DEGs), and lipid droplet (670 DEGs) were prominent (**Figure 2A**).

For up-regulated DEGs, ATP-binding and defense response were the primary subcategories (**Figure 2B**). For down-regulated DEGs (**Figure 2C**), extracellular region, membrane and apoplas were notable in CC; oxylipin biosynthetic process, defense response, and green leaf volatile biosynthetic process were key in BP; heme binding, transcription cis-regulatory region binding and xyloglucan:xyloglucosyl transferase activity were abundant in MF.

**Figure 3:**
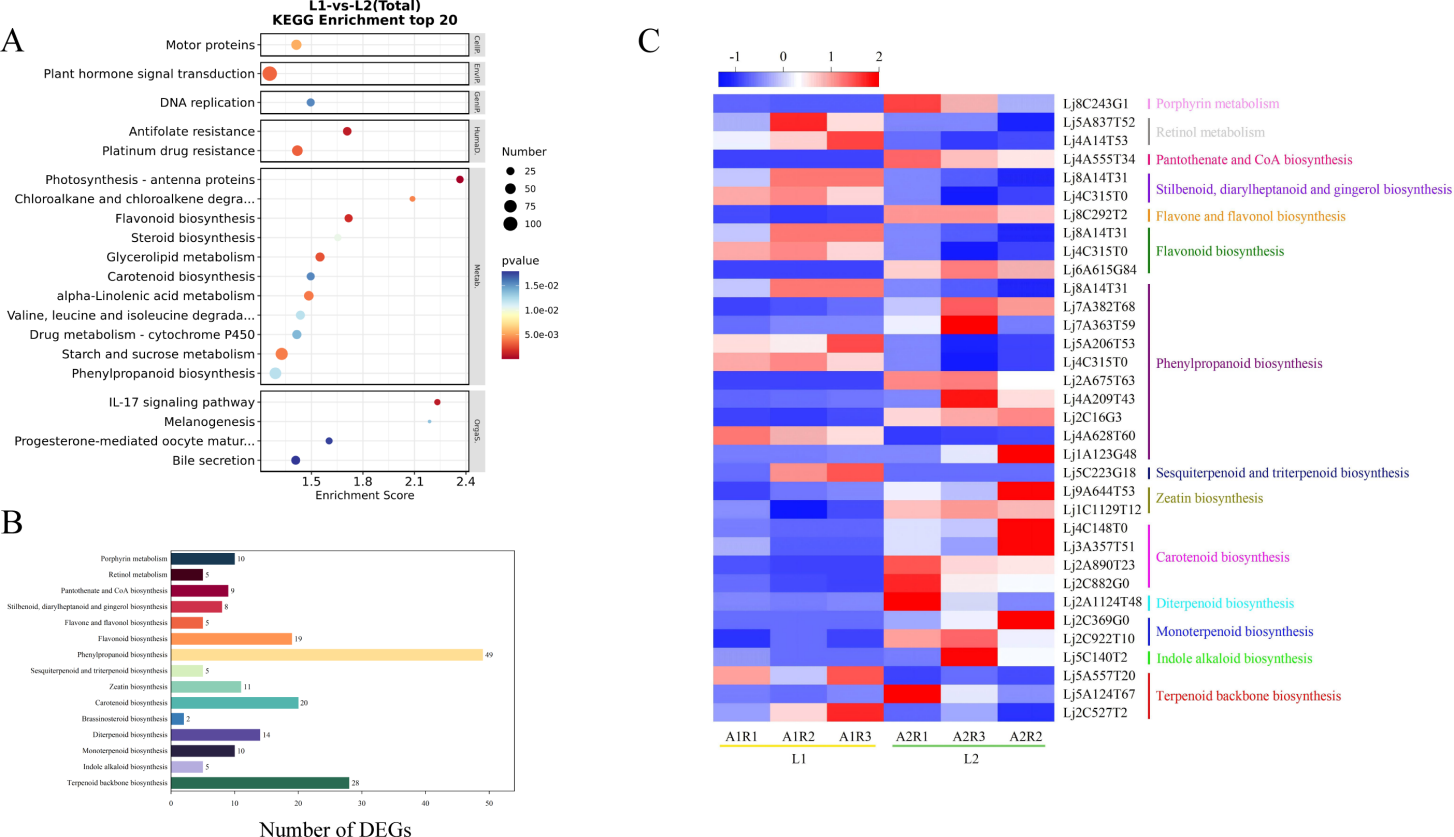
**(A)** KEGG analysis of DEGs, **(B)** The KEGG pathways of DEGs related to bioactive ingredients metabolism, **(C)** Heatmap of 34 DEGs related to metabolism of active ingredients.

KEGG analysis annotated 112 pathways, with significant enrichment in metabolic pathways, carbohydrate metabolism, amino acid metabolism, lipid metabolism, secondary metabolite biosynthesis, terpenoid and polyketide metabolism, and xenobiotics biodegradation. Notably, the 20 most enriched pathways included phenylpropanoid biosynthesis, alpha-linolenic acid metabolism, flavonoid biosynthesis, terpenoid backbone biosynthesis, and carotenoid biosynthesis (**Figure 3A**). These results suggest that DEGs may influence bioactive compound accumulation in flowers.

### 3.3 Analysis of DEGs related to the metabolism of bioactive ingredients

A total of 112 metabolic pathways were identified in the KEGG database, including 26 pathways related to bioactive compound metabolism. Among these, phenylpropanoid biosynthesis had the most DEGs (187), followed by flavonoid metabolism (66 DEGs) and vitamin metabolism (45 DEGs). The terpenoid metabolic pathway, which includes backbone, monoterpene, diterpene, brassinosteroid, carotenoid, zeatin, and sesquiterpene/triterpene biosynthesis, contained 90 DEGs (**Figure 3B**). These results suggest that many candidate genes are involved in the metabolism of bioactive compounds such as flavonoids and terpenoids in flowers.

Based on KEGG annotation and NR/PubMed databases, 34 DEGs related to bioactive compound metabolism were identified (**Table 1**). Transcriptome FPKM analysis (**Figure 3C**) showed that compared to ‘Honghua’, ‘Luyu No. 1’ had higher expression of 13 genes and lower expression of 21 genes. Key enzymes included four for carotenoid biosynthesis (abscisic-aldehyde oxidase, aldehyde oxidase 4, 15-cis-zeta-carotene isomerase, abscisic acid 8’-hydroxylase CYP707A1) and two for zeatin biosynthesis (zeatin O-glucosyltransferase, cytokinin dehydrogenase 6). One gene each was identified for monoterpenoid, diterpenoid, indole alkaloid, pantothenate/CoA, and flavone/flavonol biosynthesis. Notably, all these DEGs were downregulated in ‘Luyu No. 1’.

**Table 1:** DEGs related to bioactive ingredients metabolism.

Gene code	NR annotation	Relative expression	Gene function
Lj2C527T2	Probable 1-deoxy-D-xylulose-5-phosphate synthase 2	up	Terpenoid backbone biosynthesis
Lj5A124T67	1-deoxy-D-xylulose 5-phosphate 2-reductoisomerase	down	
Lj5A557T20	Mevalonate kinase	up	
Lj5C140T2	Strictosidine synthase	down	Indole alkaloid biosynthesis
Lj2C922T10	(+)-neomenthol dehydrogenase	down	Monoterpenoid biosynthesis
Lj2C369G0	(-)-alpha-terpineol synthase	down	Diterpenoid biosynthesis
Lj2A1124T48	Xanthotoxin 5-hydroxylase CYP82C4	down	
Lj2C882G0	Abscisic-aldehyde oxidase	down	Carotenoid biosynthesis
Lj2A890T23	Aldehyde oxidase 4	down	
Lj3A357T51	15-cis-zeta-carotene isomerase	down	
Lj4C148T0	Abscisic acid 8’-hydroxylase CYP707A1	down	
Lj1C1129T12	Zeatin O-glucosyltransferase	down	Zeatin biosynthesis
Lj9A644T53	Cytokinin dehydrogenase 6	down	
Lj5C223G18	Squalene monooxygenase SE1	up	Sesquiterpenoid and triterpenoid biosynthesis
Lj1A123G48	Peroxidase 4	down	Phenylpropanoid biosynthesis
Lj4A628T60	Caffeic acid 3-O-methyltransferase	up	Flavonoid biosynthesis
Lj2C16G3	Phenylalanine ammonia-lyase	down	
Lj4A209T43	Peroxidase 72	down	
Lj2A675T63	Peroxidase 15	down	
Lj4C315T0	Probable caffeoyl-CoA O-methyltransferase	up	
Lj5A206T53	Peroxidase 10	up	
Lj7A363T59	Peroxidase 43	down	
Lj7A382T68	Peroxidase 12	down	
Lj8A14T31	Acyltransferase GLAUCE	up	
Lj6A615G84	Chalcone synthase 1	down	
Lj4C315T0	Probable caffeoyl-CoA O-methyltransferase	up	
Lj8A14T31	Acyltransferase GLAUCE	up	
Lj8C292T2	Anthocyanidin-3-O-glucoside rhamnosyltransferase	down	Flavone and flavonol biosynthesis
Lj4C315T0	Probable caffeoyl-CoA O-methyltransferase	up	Stilbenoid, diarylheptanoid and gingerol biosynthesis
Lj8A14T31	Acyltransferase GLAUCE	up	Pantothenate and CoA biosynthesis
Lj4A555T34	Ketol-acid reductoisomerase	down	Retinol metabolism
Lj4A14T53	Alcohol dehydrogenase-like 7	up	
Lj5A837T52	8-hydroxygeraniol oxidoreductase	up	
Lj8C243G1	Protein STAY-GREEN homolog	down	Porphyrin metabolism

Additionally, 49 genes encoding 10 enzymes in the phenylpropanoid biosynthesis pathway were identified, including peroxidases (4, 72, 15, 10, 43, 12), caffeic acid 3-O-methyltransferase, phenylalanine ammonia-lyase, caffeoyl-coao-methyltransfer ase, and acyltransferase GLAUCE. Other notable genes included three for terpenoid backbone biosynthesis, three for flavonoid biosynthesis, two for porphyrin metabolism, two for stilbenoid/diarylheptanoid/gingerol biosynthesis, and one for sesquiterpene/triterpene biosynthesis and retinol metabolism. Genes involved in stilbenoid/diarylheptanoid/gingerol biosynthesis, retinol metabolism, and sesquiterpene/triterpene biosynthesis were upregulated in ‘Luyu No. 1’ (**Figure 3C**).

### 3.4 Analysis of transcription factors related to bioactive ingredient metabolism

**Figure 4:**
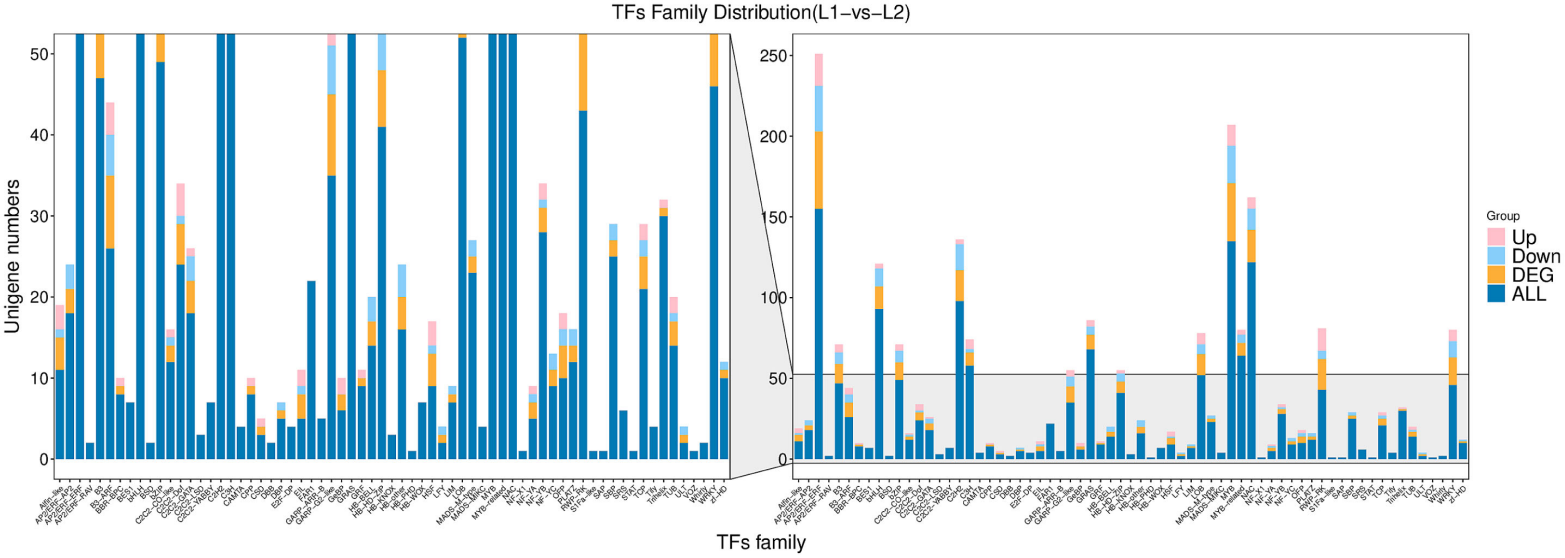
Transcription factors family distribution of ‘Honghua’ vs ‘Luyu No.1’.

Transcription factors (TFs) regulate plant metabolism, growth, development, and stress responses by modulating gene expression. This study identifies TFs involved in the biosynthesis of active components in honeysuckle by analyzing transcriptome data from ‘Luyu No. 1’ and ‘Honghua’, providing insights into their mechanisms. In the transcriptome data, 1,586 unigenes were annotated as TFs, representing 67 families (**Figure 4**).

The top 10 families include AP2/ERF-ERF (155), MYB (135), NAC (122), C2H2 (98), bHLH (93), GRAS (68), MYB-related (64), C3H (58), LOB (52), and bZIP (49). Enrichment analysis showed that 27 TF families are involved in flavonoid biosynthesis. Among these, seven differentially expressed TFs were identified: MYB3 (Lj4A178G43), MYB4 (Lj3A874T74), MYB20 (Lj3A1064T98), EOBII (Lj7A593T47, Lju41A37T46), NAC078 (Lju124C9T3), and TCP15 (bHLH Lj9C462T10). Compared to ‘Luyu No. 1’, TCP15 was significantly downregulated in ‘Honghua’, while other TFs were upregulated (**Figure 4**).

### 3.5 Analysis of metabolome between ‘Luyu No. 1’ and ‘Honghua’

**Figure 5:**
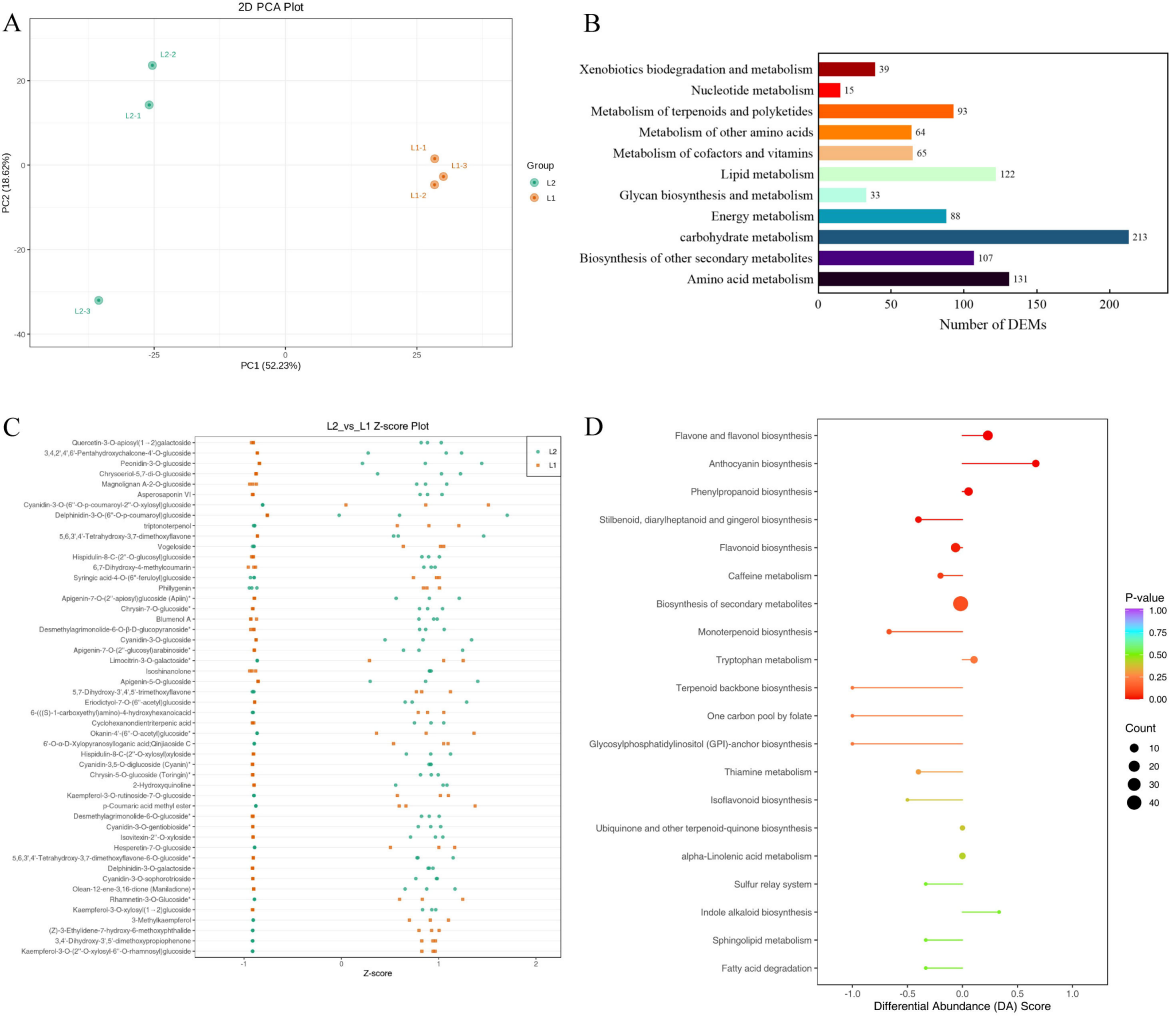
Metabolome analysis of ‘Honghua’ and ‘Luyu No.1’ flower in Dabai stage. **(A)** Scores scatter plot of the flower metabolome at Dabai stage as determined by PCA, **(B)** The KEGG pathways related to bioactive ingredients metabolism, **(C)** The distribution of differential metabolites among different groups, **(D)** Comprehensive analysis of global alterations in metabolic pathways.

Metabolome analysis identified 1,950 differentially available metabolites (DAMs) (**Supplementary 3**). Samples from ‘Luyu No. 1’ and ‘Honghua’ showed distinct clustering via Pearson’s correlation coefficient-based PLS-DA, with significant inter-group separation and good intra-group reproducibility confirmed by quality control samples (**Figure 5A**). The metabolites were predominantly flavonoids, phenolic acids, terpenoids, lignans, coumarins, amino acids and derivatives, alkaloids, lipids, quinones, organic acids, nucleotides and derivatives, steroids, tannins, and others (**Figures 5B, C**).

**Figure 6:**
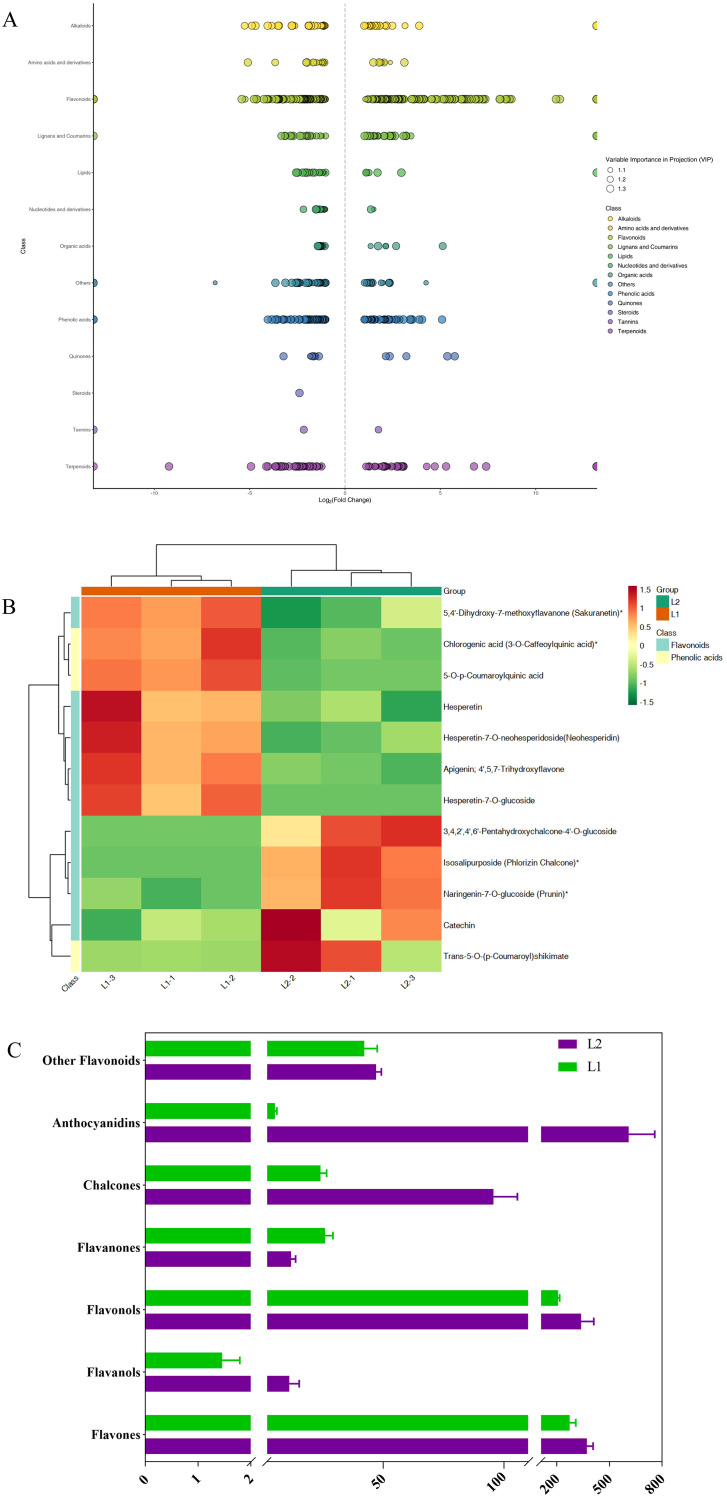
**(A)** Scatter plot of differential metabolites, **(B)** Clustered heatmap of differential metabolites in the ko00940 pathway, **(C)** Relative content of different flavonoid categories, L1: ‘Luyu No.1’; L2: ‘Honghua’.

Compared to ‘Luyu No. 1’, ‘Honghua’ exhibited up-regulation of 307 DAMs and down-regulation of 368 DAMs (**Supplementary 3**), with many belonging to phenolic acids and flavonoids (**Figure 5D**). These DAMs were enriched in pathways such as flavone/flavonol biosynthesis (**Figure 6A**), anthocyanin biosynthesis, phenylpropanoid biosynthesis (**Figure 6B**, flavonoid biosynthesis, and secondary metabolite biosynthesis, **Table 2**).

**Table 2:** Common enriched pathway of DEGs and DEMs between Honghua vs Luyu No. 1.

Comparison	KEGG ID	Pathway
Honghua vs Luyu No. 1	ko00592	alpha-Linolenic acid metabolism
	ko00940	Phenylpropanoid biosynthesis
	ko04075	Plant hormone signal transduction
	ko00941	Flavonoid biosynthesis
	ko00944	Flavone and flavonol biosynthesis
	ko00900	Terpenoid backbone biosynthesis
	ko02010	ABC transporters
	ko00902	Monoterpenoid biosynthesis
	ko00350	Tyrosine metabolism
	ko00053	Ascorbate and aldarate metabolism
	ko00563	Glycosylphosphatidylinositol (GPI)-anchor biosynthesis
	ko00901	Indole alkaloid biosynthesis
	ko00380	Tryptophan metabolism
	ko00410	beta-Alanine metabolism
	ko00040	Pentose and glucuronate interconversions
	ko00670	One carbon pool by folate
	ko00130	Ubiquinone and other terpenoid-quinone biosynthesis
	ko00950	Isoquinoline alkaloid biosynthesis
	ko00260	Glycine, serine and threonine metabolism
	ko04122	Sulfur relay system
	ko00290	Valine, leucine and isoleucine biosynthesis
	ko00730	Thiamine metabolism
	ko00908	Zeatin biosynthesis
	ko00945	Stilbenoid, diarylheptanoid and gingerol biosynthesis
	ko00360	Phenylalanine metabolism
	ko00564	Glycerophospholipid metabolism
	ko00330	Arginine and proline metabolism
	ko00770	Pantothenate and CoA biosynthesis
	ko00999	Biosynthesis of various plant secondary metabolites
	ko00520	Amino sugar and nucleotide sugar metabolism
	ko00030	Pentose phosphate pathway
	ko00740	Riboflavin metabolism
	ko00071	Fatty acid degradation
	ko00620	Pyruvate metabolism
	ko00310	Lysine degradation
	ko00650	Butanoate metabolism
	ko00660	C5-Branched dibasic acid metabolism
	ko00960	Tropane, piperidine and pyridine alkaloid biosynthesis
	ko00760	Nicotinate and nicotinamide metabolism
	ko00300	Lysine biosynthesis
	ko00230	Purine metabolism
	ko00970	Aminoacyl-tRNA biosynthesis
	ko00240	Pyrimidine metabolism
	ko00600	Sphingolipid metabolism
	ko00190	Oxidative phosphorylation

Flavonoids are the primary secondary metabolites in honeysuckle, with 227 detected in this study. As shown in **Figure 6C**, ‘Honghua’ had 2.32 times the total flavonoid content of ‘Luyu No. 1’. Flavonoids were classified into flavones, flavanols, flavonols, flavanones, chalcones, anthocyanins, and others. Comparative analysis revealed higher levels of flavones, flavanols, anthocyanidins, chalcones, and flavonols in ‘Honghua’. Specifically, anthocyanidins, flavanols, and chalcones were 87.58 times, 7.66 times, and 6.17 times higher, respectively (**Figure 6C**).

### 3.6 Integrated transcriptome and targeted-metabolome analysis

**Figure 7:**
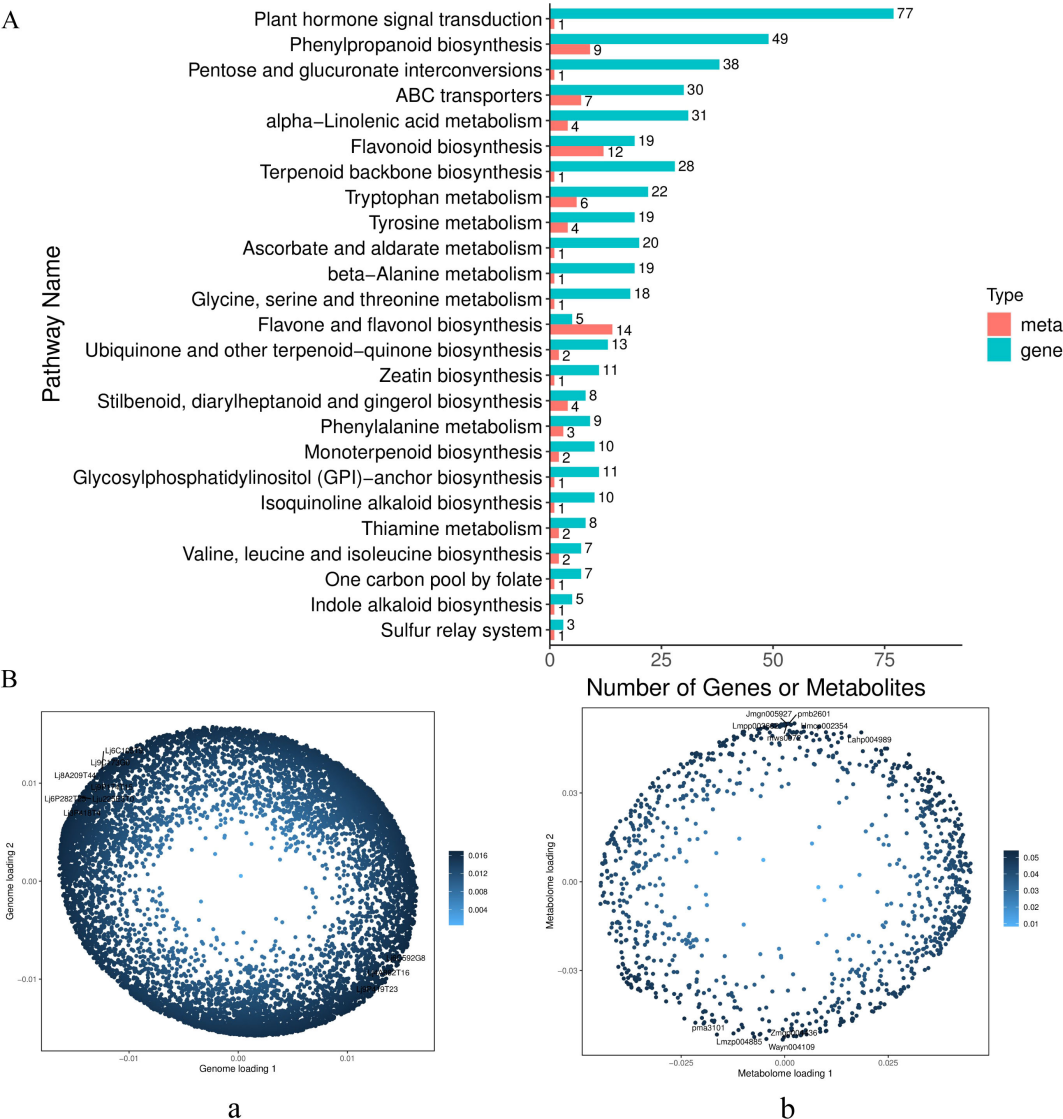
Transcriptome-metabolome correlation analysis. **(A)** KEGG enrichment correlation analysis of Honghua vs Luyu No.1 **(B)** Left, transcriptome loading plot (a) (The first ten load gene IDs Lj9P419T23, Lj6C592G8, Lj8A209T44, Lj6P282T25, Lj9C173G0, Lj6C105T9, Lju225E3T0, Lj3P418T4, Lj9P174T15, Lj4A882T16); right, metabolome loading plot (b) (The first ten load meta IDs pma3101, Lahp004989, Hmcp002354, pmb2601, Jmgn005927, Wayn004109, Zmgp004436, Lmzp004885, mws0072).

To elucidate the differences between ‘Luyu No. 1’ and ‘Honghua’, an integrated analysis of gene expression and metabolite accumulation was performed. Differentially expressed genes (DEGs) and metabolites (DEMs) were mapped to KEGG pathways, revealing 25 significantly enriched pathways in Honghua compared to Luyu No. 1 (**Figure 7A**).

For further insights, Orthogonal Projections to Latent Structures (O2PLS) modeling was applied to identify variables with high correlations across datasets. The loadings plots highlighted significant variables influencing phenological indicators. The distance from the origin or bar height represented the strength of correlation, with darker colors indicating stronger correlations. Among the top 10 substances influencing other omics layers, six flavonoids were identified: 3,7,3’-tri-O- methylquercetin-4’-O-β-D-apiofuranosyl-(1→2)-O-β-D-glucopyranoside (Lahp004989), 2-hydroxynaringenin (Jmgn005927), okanin-4’-(6’’-O-acetyl) glucoside (Zmgp004436), tricin (Lmzp004885), apigenin-5-O-glucoside (mws0072), and delphinidin-3-O-(6’’-O-p-coumaroyl) glucoside (Lmpp003662) (**Figure 7B**).

**Figure 8:**
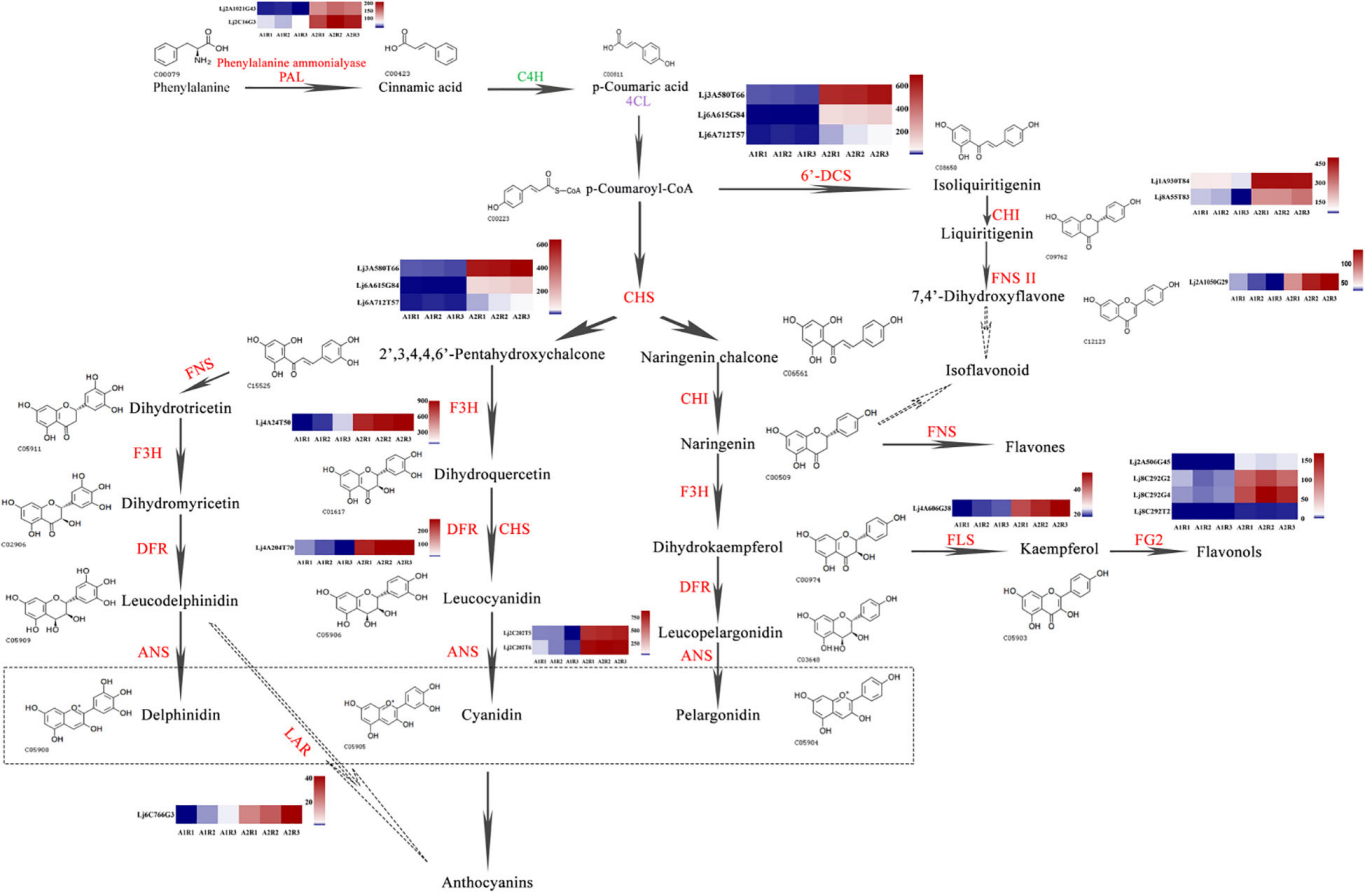
Schematic representation of the favonoid biosynthesis pathway coupled with heatmaps of relevant genes involved in the pathway. The enzyme names are abbreviated as follows: PAL, phenylalanine ammonialyase; C4H, cinnamic acid 4-hydroxylase; 4CL, 4-coumarate: CoA ligase; CHS, chalcone synthase; CHI, chalcone isomerase; FNS, favone synthase; FNSII, flavone synthaseII; F3H, favanone 3-hydroxylase; 6-DCS, 6-deoxychalcone synthase; F3’H, favonoid 3-hydroxylase; FLS, favonol synthase; DFR, dihydrofavonol 4-reductase; ANS, anthocyanidin synthase; LAR, leucoanthocyanidin reductase.

Given the significant differences observed, flavonoid biosynthesis was selected as a focal point for further investigation. Correlation analysis identified key metabolites and genes associated with flavonoid synthesis. Using a significance threshold of P < 0.05, 21 DEGs and 22 DEMs were found to be significantly associated (**Figure 8**). Specifically, among these, 20 DEGs were upregulated, 1 downregulated, 4 metabolites were upregulated, and 7 downregulated (**Supplementary 3**).

### 3.7 Analysis of differentially expressed genes related to flavonoid biosynthesis in ‘Luyu No. 1’ and ‘Honghua’

Flavonoids, the major bioactive components of honeysuckle, exhibit a range of beneficial biological and pharmacological properties. Phenylalanine, the precursor of flavonoid biosynthesis, is converted into p-coumaroyl-CoA via phenylalanine ammonia-lyase (PAL) and subsequently undergoes further enzymatic transformations to produce various flavonoid classes, including flavones, isoflavonoids, flavonols, and anthocyanins.

Specifically, p-coumaroyl-CoA is catalyzed by chalcone synthase (CHS) to form 2’,3,4,4’,6’-pentahydroxychalcone and naringenin chalcone. Naringenin chalcone is then acted upon by chalcone isomerase (CHI) to produce naringenin, which can be further metabolized into flavones by flavone synthase (FNS), or into dihydrokaempferol by flavanone 3-hydroxylase (F3H). Dihydrokaempferol is subsequently transformed into flavonols by flavonol synthase (FLS) and flavonol-3-O-glucoside L-rhamnosyltransferase. Additionally, p-coumaroyl-CoA can also serve as a precursor for isoliquiritigenin, leading to the synthesis of isoflavonoids.

In Honghua, genes involved in flavonoid biosynthesis, such as PAL, CHI, CHS, FNS, F3H, dihydroflavonol 4-reductase (DFR), anthocyanidin synthase (ANS), and flavonol glycosyltransferase (FG2), exhibited significant upregulation compared to Luyu No. 1 (**Figure 8**). Given that p-coumaric acid serves as a key precursor for flavonoid biosynthesis, our findings suggest that variations in p-coumaric acid levels may significantly influence flavonoid synthesis in different honeysuckle varieties.

### 3.8 Integrated metabolome and transcriptome analysis of flavonoid differences between ‘Luyu No. 1’ and ‘Honghua’

**Figure 9:**
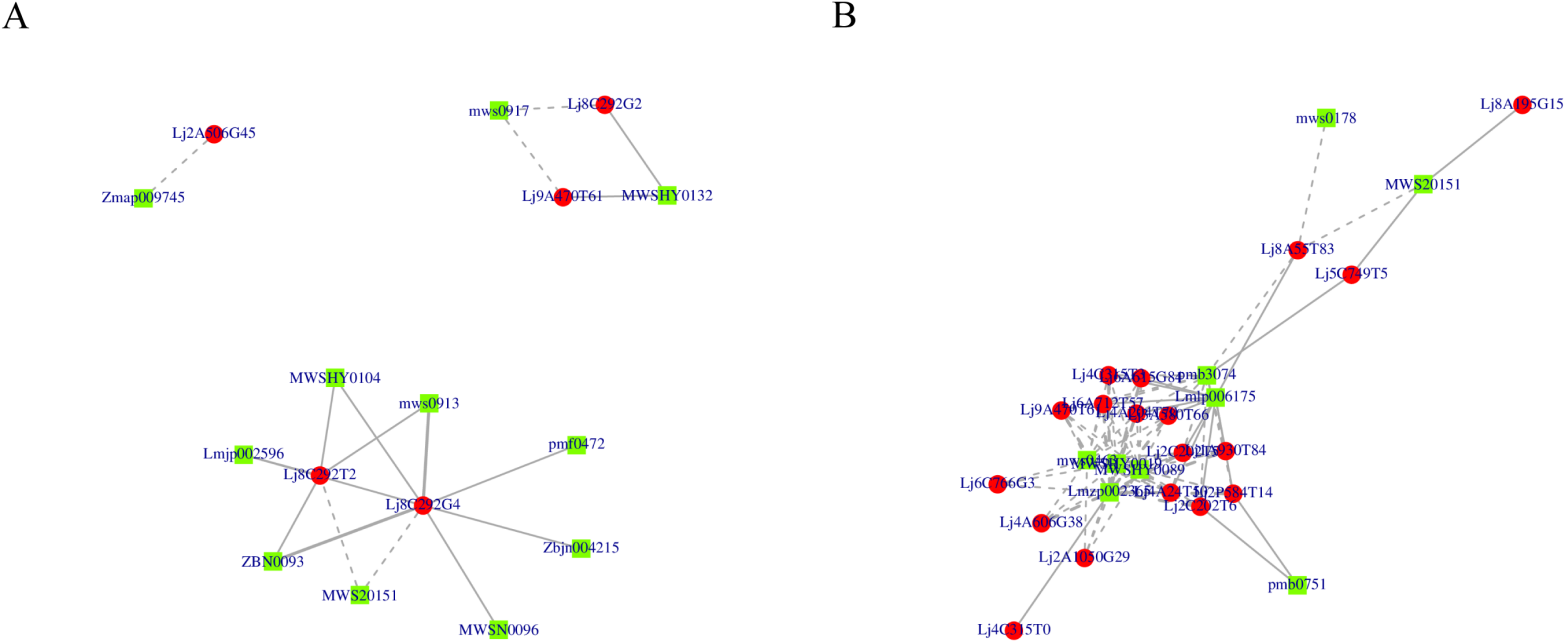
Correlation analysis of metabolites related to flavonoid synthesis-related genes. **(A)** Correlation analysisof the contents of aayafolin, 3,7-di-0-methylquercetin and apigenin with the genes related toflavonoid synthesis. **(B)**Correlation analysis of the contents of quercetin-3-0-rutinoside, quercetin-3.-rhamnoside, quercetin-3-0-sambubioside, kaempferol-3-0-galactoside, kaempferol-3-0rhamnoside, kaempferol-3-0-rutinoside, apigenin-7-0-(2"-apiosyl) glucoside and luteolin-7-0.glucoside with the genes related to flavonoid synthesis.

The primary flavonoid differential metabolites identified in honeysuckle varieties (**Figure 9**.) include aayafolin, 3,7-di-O-methylquercetin, quercetin-3-O-rhamnoside, luteolin-7-O-glucoside, apigenin, quercetin-3-O-robinobioside, kaempferol-3-O- galactoside, apigenin-7-O-(2’’-apiosyl) glucoside, kaempferol-3-O-robinobioside, kaempferol-3-O-robinotrioside, and quercetin-3-O-sambubioside. Notably, aayafolin, 3,7-di-O-methylquercetin, and apigenin are more abundant in ‘Luyu No. 1’, whereas quercetin-3-O-rhamnoside, luteolin-7-O-glucoside, quercetin-3-O-robinobioside, kaempferol-3-O-galactoside, apigenin-7-O-(2’’-apiosyl) glucoside, kaempferol-3-O- robinobioside, kaempferol-3-O-robinotrioside, and quercetin-3-O-sambubioside are more abundant in ‘Honghua’.

The differences in flavonoid content among varieties can be attributed to the differential expression of biosynthetic genes. Specifically, the expression levels of *Lj2A506G45*, *Lj8C292G2*, *Lj8C292T2*, *Lj8C292G4*, and *Lj9A470T61* influence the activities of anthocyanin-3-O-glucoside rhamnosyltransferase and flavonoid 3’-monooxygenase. In ‘Honghua’, higher expression of *Lj2A56G45*, *Lj9A470T61*, *Lj8C292T2*, and *Lj8C292G4* negatively correlates with the levels of aayafolin, 3,7-di-O-methylquercetin, and apigenin, leading to their higher abundance in ‘Luyu No. 1’ (**Figure 9A**).

Conversely, elevated expression of *Lj8C292G2* and *Lj9A470T61* positively correlates with quercetin-3-O-rutinoside levels in ‘Honghua’, enhancing the activities of anthocyanin-3-O-glucoside rhamnosyltransferase and flavone 3’-monooxygenase, which results in higher quercetin-3-O-rutinoside levels in ‘Honghua’ compared to ‘Luyu No. 1’. Additionally, high expression of *Lj8C292T2* and *Lj8C292G4* in ‘Honghua’ increases the activity of anthocyanin-3-O-glucoside rhamnosyltransferase, contributing to higher levels of luteolin-7-O-glucoside, quercetin-3-O-rhamnoside, kaempferol-3-O-galactoside, apigenin-7-O-(2’’-apiosyl) glucoside, kaempferol-3 -O-rhamnoside, kaempferol-3-O-rutinoside, and quercetin-3-O-sambubioside in ‘Honghua’ (**Figure 9B**).

### 3.9 Quantitative Real-time PCR validation of gene expression profiles

**Figure 10:**
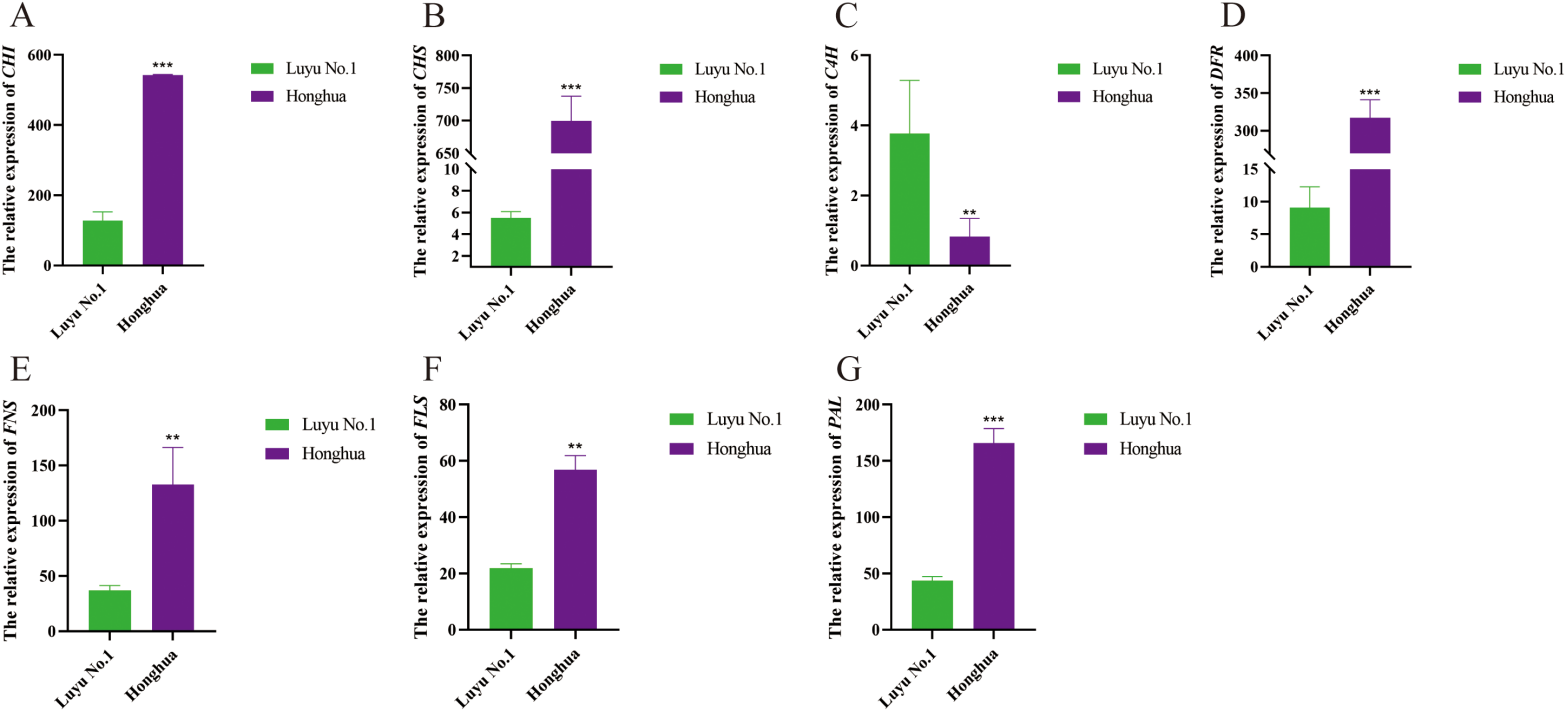
Relative expression levels of three transcription factors in different tissues of ‘Honghua’ and ‘Luyu No.1’. **(A)** CHI, **(B)** CHS, **(C)** C4H, **(D)** DFR, **(E)** FNS, **(F)** FLS, **(G)** PAL. Asterisks’*’ represent statistical differences in the same index between different species, with p<0.05 being a significant difference (**p<0.01, ***p<0.001).

Eight representative differentially expressed genes (DEGs) from ‘Luyu No. 1’ and ‘Honghua’ were selected for RT-qPCR validation. The selection criterion was based on genes exhibiting an expression level at least twice that of the other variety at this stage. The validation results showed a high degree of consistency with the transcriptome data (**Figure 10**), confirming the reliability of the transcriptome analysis. The eight candidate genes exhibited two predominant expression patterns. Specifically, the expression levels of chalcone synthase (*Lj3A580T66*), chalcone isomerase (*Lj1A930T84*), flavone synthase (*Lj2A1050G29*), flavonol synthase (*Lj4A606G38*), bifunctional dihydroflavonol 4-reductase (*Lj4A204T70*), and phenylalanine ammonia-lyase (*Lj2A1021G43*) were significantly upregulated in ‘Honghua’. In contrast, the expression of cinnamate 4-hydroxylase 3 (*Lj5C749T5*) was down- regulated in’ Honghua’.

## 4 Discussion

Honeysuckle, a traditional medicinal herb and food supplement, is renowned for its rich content of bioactive compounds, including flavonoids, volatile oils, organic acids, saponins, and iridoid glycosides (Lee et al., 2021). These compounds endow honeysuckle with significant antiviral properties, contributing to its heat-clearing and detoxification effects, as well as its involvement in inflammation treatment and antitumor processes (Muro et al., 2025). Among these bioactive compounds, flavonoids are particularly noteworthy. They are derived from flavone (2-phenylchromene) and include its isomers and hydrogenated reduction products. As plant secondary metabolites, flavonoids predominantly exist as glycosides or carbon glycosyls combined with sugars, although some occur in free form (Okom et al., 2025). Flavonoids are the major bioactive components in honeysuckle, and they exhibit a range of beneficial biological and pharmacological properties (Guo et al., 2025). For instance, flavonoids isolated from honeysuckle buds have been shown to inhibit the respiratory syncytial virus and effectively treat respiratory viral infections (Xu et al., 2024). Additionally, these flavonoids can suppress the release of inflammatory mediators such as TNF-α and IL-6, thereby alleviating inflammatory responses (Lu et al., 2024).

Previous studies have identified a wide variety of flavonoids in different plant species. For example, 37 differential flavonoids have been identified in tomatoes using UPLC-MS/MS, 41 flavonoids in kiwifruit (with 7 differing significantly between two varieties) (Liu et al., 2025), and 254 flavonoids in five citrus varieties (with 169 differentially accumulated) (Cao et al., 2023). Research on flavonoid metabolomics has predominantly focused on anthocyanin biosynthesis in differently colored varieties, such as figs (Mao et al., 2024), Many flower Solomon seal (Fan et al., 2023), and sweet potatoes (Sun et al., 2025), where significant variations in anthocyanin composition have been observed. In the present study, a comprehensive metabolomic analysis of Dabai-period buds from two honeysuckle varieties, ‘Luyu No. 1’ and ‘Honghua,’ revealed the presence of 399 flavonoids, including 19 chalcones, 161 flavones, 143 flavonols, 17 flavanols, 13 flavanones, 27 anthocyanidins, and 14 others. Notably, 228 flavonoids differed between the two varieties, specifically 14 chalcones, 80 flavones, 75 flavonols, 6 flavanols, 21 flavanones, 20 anthocyanidins, and 11 others. These differences in flavonoid abundance likely contribute to the quality variations observed among honeysuckle varieties.

The biosynthetic pathway of flavonoids involves numerous structural and regulatory genes (Yang et al., 2024). In this study, 5901 differentially expressed genes (DEGs) were identified during the Dabai-period bud development of ‘Luyu No. 1’ and ‘Honghua’ using Illumina sequencing. Functional annotation revealed transcripts encoding genes involved in bioactive compound metabolism. Combining annotations from the NR and PubMed databases, 34 DEGs related to flavonoid, carotenoid, monoterpenoid, alkaloid, and vitamin metabolic pathways were identified. Specifically, 18 DEGs encoding 11 enzymes, including PAL, CHS, CHI, F3H, FLS, flavone synthase II, FG2, ANS, DFR, and LAR, were found to be involved in flavonoid biosynthesis. Among these, one gene was downregulated, while 20 were upregulated in ‘Honghua’.

These findings demonstrate a strong correlation and functional consistency with the observation that the total flavonoid content in ‘Honghua’ is 2.32-fold higher than that in ‘Luyu No. 1’. This suggests that the upregulated genes may actively enhance flavonoid accumulation, whereas the downregulated gene could serve as a limiting factor in ‘Luyu No. 1’. The identification of these genes provides key candidate targets for elucidating the molecular mechanisms underlying flavonoid biosynthesis in the two cultivars and facilitates the development of high-flavonoid honeysuckle varieties with improved medicinal value.

Notably, the flavonoid pathway produces multiple enzyme metabolites, including anthocyanins (Zhao et al., 2022). Since these compounds share an upstream pathway, the DEGs are also involved in flavone, isoflavone, and flavonol metabolism. In this study, the isoflavone content was lower in ‘Luyu No. 1’ than in ‘Honghua,’ while flavanone levels were significantly higher in ‘Luyu No. 1.’ This difference may result from the differential expression of pivotal genes such as *PAL*, *4CL*, *CHS* and *FNS*. Specifically, PAL is the first enzyme in phenylpropanoid biosynthesis, making its expression crucial for flavonoid biosynthesis (Chen et al., 2024). *PAL* expression was considerably higher in ‘Honghua,’ making it a key candidate gene for studying flavonoid and isoflavone differences between the varieties.

The regulation of flavonoid synthesis in different varieties primarily occurs at the transcriptional level. Transcription factors such as bHLH, MYB, and EOBII are involved in this process (Chen et al., 2025). In this study, several transcription factors were differentially expressed: *MYB* (*MYB3, MYB4, MYB20, EOBII*), *NAC078*, and *bHLH* (*TCP15*). Notably, *MYB3*, *MYB4*, and *MYB20* were all downregulated in ‘Honghua.’ *Lj3A874T74*, a gene closely related to *MYB*4 (a well-characterized repressor of flavonoid biosynthesis genes in other plant species) was significantly downregulated in ‘Honghua’ (Soostani et al., 2025). This downregulation may alleviate the transcriptional repression of key flavonoid biosynthetic enzymes, thereby contributing to the elevated flavonoid accumulation observed in this variety. Conversely, the sustained expression of *Lj3A874T74* in ‘Luyu No. 1’ may partially account for the reduced flavonoid content in that variety.

EOBII is a member of the MYB transcription factor family that plays a regulatory role in anthocyanin and flavonol metabolism (Qi et al., 2021; Aharoni et al., 2001). Functionally characterized homologs of EOBII, including *Antirrhinum majus MYB305/MYB340* and *Nicotiana NlxNs MYB305*, have been shown to directly activate genes within the flavonoid biosynthetic pathway, particularly those involved in flavonol glycosylation (Yang et al., 2025; Liu et al., 2021). The upregulation of *Lj7A593T47* and *Lju41A37T46*—identified as EOBII homologs—in ‘Honghua’ suggests their potential involvement in promoting flavonol biosynthesis in honeysuckle, which is consistent with the observed accumulation of specific flavonol content in this variety.

Furthermore, the pronounced upregulation of *TCP15* (bHLH *Lj9C462T10*) in ‘Honghua’ is particularly noteworthy. bHLH transcription factors are well-established regulators that commonly form heterodimeric complexes with MYB transcription factors to activate the expression of late-stage biosynthetic genes within the flavonoid and anthocyanin biosynthetic pathways (Baudry et al., 2004). Functionally characterized homologs such as *CsbHLH62* (involved in catechin biosynthesis in tea), AtGL3/EGL3 (regulating flavonoid biosynthesis in *Arabidopsis*), *SlAN1* (associated with anthocyanin accumulation in tomato), *CmbHLH2* (linked to anthocyanin biosynthesis in *chrysanthemum*), and *AtTT8* (a key regulator of anthocyanin gene expression in *Arabidopsis*) collectively illustrate the evolutionarily conserved role of this transcription factor family in promoting flavonoid biosynthesis (Castillejo et al., 2020; Deng et al., 2022; Liu et al., 2023; Hong et al., 2021). The elevated expression level of *TCP15* in ‘Honghua’ strongly implies its involvement in enhancing flavonoid biosynthesis, with likely contributions to anthocyanin accumulation in this red-pink-flowered cultivar. Nevertheless, definitive elucidation of its specific target genes and interacting partners—particularly MYB cofactors—requires further functional characterization in honeysuckle.

Among the structural genes involved in flavonoid biosynthesis, the differential expression patterns of those encoding anthocyanidin-3-O-glucoside rhamnosyl- transferase (3RT) and flavonoid 3’-monooxygenase (F3’M) merit in-depth functional analysis due to their significant influence on flavonoid chemistry and pigmentation. Specifically, 3RT catalyzes the transfer of a rhamnose moiety to the glucose residue at the 3-hydroxyl position of anthocyanidins. This glycosylation event is critically important as it markedly enhances the stability and water solubility of anthocyanins (Li et al., 2022), thereby directly influencing pigment accumulation and color intensity (Kroon et al., 1994). Functional studies in *Petunia hybrida* have demonstrated that loss-of-function mutations in the 3RT gene (*Rt*) result in a pronounced shift in flower color from blue-purple to pink, primarily due to the reduced stability of unmodified anthocyanin molecules (Kroon et al., 1994). In our study, the coordinated and significantly elevated expression of multiple 3RT genes (*Lj8C292G2*, *Lj8C292T2*, *Lj8C292G4*, *Lj2A506G45*) in ‘Honghua’—resulting in a cumulative 22.12-fold increase in transcript levels for this enzyme class—strongly indicates that enhanced anthocyanin rhamnosylation serves as a key molecular mechanism underlying the remarkable 87.58-fold increase in anthocyanidin content and the stable, intense red-pink pigmentation characteristic of this variety.

F3’M, a cytochrome P450 enzyme commonly classified as CYP75B, catalyzes the hydroxylation of the 3’ position on the B-ring of flavonoid substrates. As a key branch-point enzyme in the flavonoid pathway, it mediates the conversion of dihydrokaempferol to dihydroquercetin and kaempferol to quercetin. This enzymatic activity determines the type of anthocyanins synthesized and significantly influences the resulting pigment hue (ranging from red to purple to blue) (Tanaka et al., 2005). The functional importance of F3’M in determining color intensity is well documented across multiple species: Wild tomato species with deep red fruit peels exhibit high F3’M activity, whereas cultivated varieties with reduced activity accumulate pelargonidin derivatives, leading to an orange-red pigmentation (Butelli et al., 2008).

Enhanced expression of *F3’M* has been shown to intensify red coloration in transgenic tomato lines. In roses, deep red cultivars such as ‘Rouge Meilland’ display higher *F3’M* expression compared to pink-flowered varieties (Schmitzer et al., 2010). Similarly, red-fleshed strawberry cultivars (‘Festival’) exhibit elevated *F3’M* levels, while white-fleshed mutants (‘White Carolina’) carry loss-of-function mutations in this gene (Edger et al., 2019). CRISPR-mediated knockout of *F3’M* in strawberries results in a shift toward orange-red pigmentation (Zhou et al., 2020), and RNAi-mediated silencing of *F3’M* in petunia leads to orange-red flowers (Fukuchi-Mizutani et al., 2003). Overexpression of *F3’M* genes in carnations transforms pale pink petals into deep red or purple hues, and overexpression of *CmF3’M* in *chrysanthemum* increases cyanidin content, shifting flower color from pink to deep red (Noda et al., 2017).

In our study, the 3.03-fold upregulation of *F3’M* (*Lj9A470T61*) in ‘Honghua’ relative to ‘Luyu No. 1’ aligns closely with the observed metabolic profile in ‘Honghua’, which includes significantly elevated levels of quercetin derivatives such as quercetin-3-O-rutinoside (rutin), quercetin-3-O-rhamnoside (hyperoside), and luteolin-7-O-glucoside—all metabolites characterized by a 3’,4’-dihydroxylated B-ring structure dependent on F3’M activity. This enhanced 3’ hydroxylation capacity likely plays a pivotal role in the intense red-pink pigmentation of ‘Honghua’ by promoting the biosynthesis of cyanidin-derived anthocyanins and specific flavon(ol) glycosides.

Integrated transcriptomic and metabolomic analyses have revealed the key role of flavonoid metabolism in mediating color formation in plant tissues, including leaves, petals, and fruits. Studies have shown that tissue-specific coloration is regulated by differential activation of flavonoid biosynthetic pathways and accumulation of secondary metabolites (Gonzalez et al., 2008). In fruit, Mou et al. (2015) identified incomplete flavonoid pathway activation as a determinant of anthocyanin patterns in tomao. Similarly, Park et al. (2016) compared three sweet potato cultivars with distinct flesh colors (white, orange, and purple), identifying seven differentially accumulated flavonoids. Their analysis highlighted MYB transcription factors, bHLH regulators, and UFGT enzymes as critical genetic factors for pigmentation differences.

Multi-omics approaches have also clarified the mechanisms of foliar coloration. Wang et al. (2022) characterized leaf color differentiation in *Docynia delavayi*, linking it to core flavonoid biosynthesis pathways. Research on *Rhododendron dauricum* showed that inter-cultivar leaf color differences arise from coordinated regulation of anthocyanin and catechin biosynthesis, with key genes clustered in flavonoid pathways (Wang et al., 2024).

Floral pigmentation studies have yielded similar findings. Cai et al. (2024) deciphered *Phyllostachys nigra* color formation, linking it to flavonoid pathways and regulatory genes. Lv et al. (2023) found that pigment formation in two brown cotton fibers cultivars result from metabolic flux redirection at the dihydroflavonol branch point, favoring anthocyanin biosynthesis. Previous studies have shown that identifying genes related to flavonoid and anthocyanin metabolism is crucial for understanding red pigment formation (Wang et al., 2022; Liu et al., 2023). Structural genes involved in flavonoid and anthocyanin biosynthesis have been identified in various plant species (Kroon et al., 1994).

In this study, integrated transcriptomic and metabolomic analyses demonstrated that the flavonoid biosynthesis pathway plays a significant role in determining honeysuckle coloration. Key upstream genes, including *PAL*, *4CL*, *CHS*, and *FNS*, were significantly upregulated, thereby ensuring an adequate supply of metabolic precursors for downstream biosynthetic processes. As previously elaborated, anthocyanidin-3-O-glucoside rhamnosyltransferase (3RT), encoded by *Lj8C292G2*, *Lj8C292T2*, *Lj8C292G4*, and *Lj2A506G45*, serves a crucial function in anthocyanin biosynthesis by enhancing pigment stability and water solubility (Li et al., 2022; Kroon et al., 1994). Similarly, flavonoid 3’-monooxygenase (F3’M), encoded by *Lj9A470T61*, represents a key regulatory enzyme in both anthocyanin and flavonol biosynthesis. It promotes the accumulation of 3’-hydroxylated flavonoids, which are associated with intensified red and purple pigmentation in plants (Butelli et al., 2008; Tanaka et al., 2005; Noda et al., 2017).

In ‘Honghua’, the expression levels of both *3RT* and *F3’M* genes were elevated by 22.12-fold and 3.03-fold, respectively, compared to ‘Luyu No. 1’. This enhanced gene expression correlates directly with their established biochemical roles and is likely responsible for the observed increases in several flavonoid glycosides, including quercetin-3-O-rutinoside (rutin), luteolin-7-O-glucoside, quercetin-3-O-rhamnoside (hyperoside), kaempferol-3-O-galactoside, apigenin-7-O-(2’’-apiofuranosyl)glucoside, kaempferol-3-O-rhamnoside, kaempferol-3-O-rutinoside, and quercetin-3-O- sophoroside, relative to ‘Luyu No. 1’. Therefore, the differential expression patterns of *3RT* and *F3’M* are strongly associated with the distinct color phenotype observed between these two honeysuckle varieties. Future research should aim to further elucidate the regulatory mechanisms governing these genes, perform functional validation studies to establish causal relationships, and explore their potential applications in breeding programs designed to enhance both the pigmentation and medicinal properties of honeysuckle.

## 5 Conclusions

In summary, integrated transcriptomic and metabolomic analyses have demonstrated that variations in genes associated with flavonoid biosynthesis and anthocyanin profiles represent key determinants of medicinal quality differences among honeysuckle varieties. A total of 228 differentially accumulated flavonoids, 11 structural genes involved in flavonoid biosynthesis, and 5 transcription factors (including MYB, bHLH, and NAC) were identified. Further analysis revealed that the expression levels of the key enzyme-encoding genes *3RT* and *F3’M* are strongly correlated with the accumulation of critical flavonoid metabolites such as quercetin-3-O-rutinoside (rutin), luteolin-7-O-glucoside, and quercetin-3-O- rhamnoside (hyperoside). These correlations are mechanistically linked to enhanced anthocyanin stability (mediated by 3RT) and B-ring hydroxylation (catalyzed by F3’M), both of which favor red pigmentation. Significant differences in flavonoid biosynthesis between the two varieties were observed, indicating regulation by a complex metabolic network. The functional roles of structural genes such as *3RT* and *F3’M*, as well as the regulatory mechanisms of the identified transcription factors (MYB, bHLH, NAC), require further experimental validation to transition from correlative observations to causal relationships. This study provides novel insights into the molecular mechanisms underlying flavonoid biosynthesis in honeysuckle and establishes a theoretical foundation for genetic improvement and metabolic engineering strategies aimed at enhancing its medicinal properties.

## Data Availability

The datasets presented in this study can be found in online repositories. The names of the repository/repositories and accession number(s) can be found in the article/**Supplementary Material**.
