# Supplementary material for: Integrated metabolomic and transcriptomic analysis elucidates transcriptional regulation of flavonoid biosynthesis in differentially pigmented honeysuckle (Lonicera japonica) varieties
Source: Front Plant Sci. 2025 Sep 11;16:1636028. doi: 10.3389/fpls.2025.1636028 (PMC12460243; doi:10.3389/fpls.2025.1636028)
Supplement: Supplementary file 1 [file Table1.doc]

**Table. S1 Primer list**

| **Gene name** | **Gene ID** | **Sequences (5’-3’)** |
| --- | --- | --- |
| Chalcone Isomerase (CHI) | Lj1A930T84 | Forward 3-‘AAGAGGAGGAAGAAGAGGCAC’-5 |
|  |  | Backward 3-‘CTCAGCAGTGGAAGAAGTTGC’-5 |
| Chalcone Synthase (CHS) | Lj3A580T66 | Forward 3-‘GCCGTTGTTTGAGGTTGTCTC’-5 |
|  |  | Backward 3-‘AGCAGATGGAAAGTAAGCCCA’-5 |
| Cinnamate 4-Hydroxylase (C4H) | Lj5C749T5 | Forward 3-‘AGTTGTTGTATCCTCGCCTGA ’-5 |
|  |  | Backward 3-‘ATCTTCCTCCAGTGCTCACC’-5 |
| Dihydroflavonol 4-Reductase Flavanone 4-Reductase (DFR) | Lj4A204T70 | Forward 3-‘CGGACCTAACAGAAGAAGGGA’-5 |
|  |  | Backward 3-‘GAGGAAGTGAAGACCAGCCT’-5 |
| Flavone Synthase (FNS) | Lj2A1050G29 | Forward 3-‘CTCATCCACCAGTCCTTCCA’-5 |
|  |  | Backward 3-‘ATGGCAGCGGAGTGTTTACG’-5 |
| Flavonol Synthase (FLS) | Lj4A606G38 | Forward 3-‘GTTCCCAACGAGGTTCAAGG’-5 |
|  |  | Backward 3-‘GCCTTGAACCTCGTTGGGAA’-5 |
| Phenylalanine Amononia-Lyase (PAL) | Lj2A1021G43 | Forward 3-‘GGAGGACTAAACAAGGCGGT’-5 |
|  |  | Backward 3-‘AAACGNATGCCNGAGTAGCC’-5 |
| Actin | KY114518 | Forward 3-‘AGGATGCTTATGTCGGCGAT’-5 |
|  |  | Backward 3-‘TGGCTTTAGGGTTGAGAGGTG’-5 |

**Table. S2 Statistics of sequencing data (A1R1, A1R2 and A1R3 are three replicates of the ‘Luyu No. 1’ flowers. A2R1, A2R2 and A2R3 are three replicates of the and ‘Honghua’ flowers.)**

| Sample | Raw reads | Clean reads | Clean bases(G) | Q30 (%) | GC content (%) | Mapping rate (%) |
| --- | --- | --- | --- | --- | --- | --- |
| A1R1 | 51.04 | 48.80 | 7.04 | 95.27 | 45.19 | 95.61 |
| A1R2 | 51.43 | 48.99 | 7.04 | 96.53 | 45.14 | 95.27 |
| A1R3 | 50.95 | 48.65 | 7.00 | 96.88 | 45.15 | 95.49 |
| A2R1 | 49.88 | 47.84 | 6.91 | 96.73 | 44.97 | 95.91 |
| A2R2 | 50.28 | 48.04 | 6.93 | 96.21 | 45.04 | 95.54 |
| A2R3 | 51.14 | 48.46 | 6.93 | 96.66 | 45.03 | 94.74 |
